# Topical Anti-Inflammatory and Analgesic Activities of *Citrullus colocynthis* Extract Cream in Rats

**DOI:** 10.3390/medicina54040051

**Published:** 2018-07-29

**Authors:** Marzieh Pashmforosh, Hossein Rajabi Vardanjani, Hassan Rajabi Vardanjani, Mahdi Pashmforosh, Mohammad Javad Khodayar

**Affiliations:** 1 Department of Pharmacology, School of Pharmacy, Student Research Committee, Ahvaz Jundishapur University of Medical Sciences, Ahvaz, Iran; Marzie_pf@yahoo.com; 2 Department of Environmental Health Engineering, School of Public Health, Shahrekord University of Medical Sciences, Shahrekord, Iran; rajabi@hlth.mui.ac.ir; 3 Faculty Member of Production Technology Research Institute, Jahad Deneshgahi, Ahvaz, Iran; m.pashmforoosh62@yahoo.com; 4 Toxicology Research Center, Ahvaz Jundishapur University of Medical Sciences, Ahvaz, Iran; jkhodayar@yahoo.com; 5 Department of Toxicology, School of Pharmacy, Ahvaz Jundishapur University of Medical Sciences, Ahvaz, Iran

**Keywords:** *Citrullus colocynthis*, cream, antinociceptive, anti-inflammatory, rat

## Abstract

*Background and objectives:Citrullus colocynthis* (CC), known as bitter apple, is used to treat diabetes in Iranian traditional medicine. The aim of this study is to evaluate the anti-inflammatory and analgesic activities of CC cream in rats. *Materials and Methods:* The carrageenan-induced edema in a rat hind paw was carried out to evaluate the topical anti-inflammatory effect of the CC fruit extract cream (2–8%) and the tissue levels of IL-6 and TNF-α were estimated by using a commercial ELISA kit. The topical antinociceptive activity of CC cream (2–8%) was evaluated in the rat formalin test. To determine the role of opioid receptors in the local antinociceptive effect of the CC cream, naloxone (20 μg/paw, i.pl.), a non-selective opioid antagonist, was used. *Results:* The results showed that the CC cream (2–8%) dose-dependently reduced the carrageenan-induced paw edema and reversed the changes in the level of TNF-α and IL-6 due to carrageenan-induced edema (*p* < 0.01). The anti-inflammatory effect of CC cream 8% was comparable to that of hydrocortisone ointment 1%. Furthermore, the application of CC cream (2–8%) dose-dependently inhibited both first and second phases of the formalin test (*p* < 0.05). The antinociceptive effect of the CC cream (8%) was comparable to that of methyl salicylate cream 30%. Moreover, the administration of naloxone significantly reversed the topical antinociceptive effect of the CC cream (*p* < 0.05). *Conclusions:* For the first time, this study indicated that the topical application of CC cream possesses significant anti-inflammatory and antinociceptive activities in animal models, which were probably mediated by opioid receptors and the suppression of pro-inflammatory cytokines (TNF-α and IL-6). Thus, the CC cream can be used to treat inflammatory pain and inflammatory diseases.

## 1. Introduction

Pain and inflammation are the most common and main symptoms of many diseases. Inflammation as the most important response of a damaged tissue induces a variety of hostile stimuli such as infections, toxic chemical agents, and physical damage resulting in redness, edema, heat, and pain [1]. Many studies have indicated that inflammatory reactions are implicated in the progression of several disorders, such as aging [1] rheumatoid arthritis [2], skin inflammation [3], and cardiovascular dysfunction [4]. The tissue inflammation is mediated by infiltration and activation of leukocytes into the damaged region and the release of pro-inflammatory mediators, including tumor necrosis factor (TNF), interleukin 6 (IL-6), and IL-1β [5]. Although non-steroidal anti-inflammatory drugs and opioids are widely used in the treatment of inflammatory diseases and pain, these drugs have a variety of side effects. The most important side effect of opioids is tolerance and the chronic administration of NSAIDs leads to gastrointestinal bleeding [6]. Therefore, research to identify anti-inflammatory and analgesic compounds with fewer side effects and more efficacy is necessary. Complementary to this, herbal compounds are one of the most important sources for the discovery of new drugs with a high safety margin [7].

*Citrullus colocynthis* (CC), known as bitter apple, is used topically to treat diabetes in Iranian traditional medicine. CC grows extensively in the center, south, and east of Iran. Most of the therapeutic effects of this plant are related to its fruit [8]. Studies have shown that the fruit of CC has a variety of pharmacological effects, including anti-inflammatory [9], analgesic [10], anticancer [11], hypolipidemia [12], immunostimulating [13], antioxidant [14], and antidiabetic [5]. The most important active ingredients of this plant are glycosides, flavonoids, alkaloids, monoterpenes, phenolic acids, triterpenoid, carbohydrates, fatty acids, and essential oils [2,5,15,16,17,18].

Previous studies have showed the systemic antinociceptive and anti-inflammatory effects of *C. colocynthis* extract, but its exact mechanisms have not been identified. Heidary et al. (2015), showed that *C. colocynthis* fruit extract possesses analgesic activity in patients with painful diabetic polyneuropathy [8]. Marzouk et al. (2010) showed that the oral administration of *C. colocynthis* fruit extract decreased paw edema induced by 1% carrageenan [2]. The protective effect of *C. colocynthis* has been attributed to the suppression of pro-inflammatory release, inhibition of COX-2 expression, and its antioxidant activity [5,10,19].

Growing evidence that systemic uses of CC cause severe adverse effects such as acute rectorrhagia, liver intoxication, abortifacient, cardio-depressant, and emetic effects, can be seen in the literature [15,20]. For instance, Javadzadeh et al. (2013) reported that the consumption of CC led to tenesmus and bloody diarrhea. The intestinal damage and rectorrhagia have been attributed to the membranolytic effect of CC constituents [21]. In addition, Dehghani et al. (2006), have shown that CC extract (˃100 mg/kg) induced hepatic intoxication, which was accompanied by hepatic fibrosis and necrosis [22]. In addition, toxic doses of CC (600–1000 mg) lead to tenesmus, hematochezia, and nephrosis. A lethal dosage (˃2 g) caused paralysis, circulatory collapse, and finally, death [15]. In sum, it is critical to save the beneficial effects of CC and limit its adverse effects with appropriate approaches.

The topical administration of drugs has many benefits, including optimizing the concentration of the drug in the position, reducing systemic drug levels, reducing systemic side effects, and reducing drug interactions. Currently, the topical form of anti-inflammatory drugs, local anesthetics, and analgesics is widely used by patients [23].

Although previous studies have shown the anti-inflammatory and analgesic activity of CC extract, its systemic use is limited due to a very bitter taste and high risk of gastrointestinal bleeding [21]. So, for the first time, in this study, we evaluated the anti-inflammatory and antinociceptive activity of the topical application of CC extract and its underlying mechanisms in animal models.

## 2. Materials and Methods

### 2.1. Chemicals

Carrageenan and formalin were purchased from Sigma-Aldrich (St. Louis, MO, USA). Hydrocortisone ointment 1%, methyl salicylate 30%, naloxone hydrochloride, and morphine sulfate (5H_2_O) were purchased from Iran Darou, Sobhan Darou Pharmaceutical Co. and Temad Pharmaceutical Co. (Tehran, Iran), respectively. ELISA kits for the determination of TNF-α and IL-6 were provided by Diaclone (Besancon, France). Solvents for extraction were of commercial grade.

### 2.2. Plant Material

*C. colocynthis* fruits used in this experiment were collected from Dezful, Khuzestan province, Iran (48°32′45.591″ N; 32°18′8.702″ E) in April 2016. These fruits were identified by the Pharmacognosy Department of Ahvaz Jundishapur University of Medical Science (AJUMS, Ahvaz, Iran), Ahvaz, Iran and were deposited at the Herbarium of the AJUMS under the label A150740102FP.

### 2.3. Hydroalcoholic Extraction

After removing the seeds, dried fruits at an ambient temperature were powdered by an electric mill. The powder was macerated with 70% aqueous ethanol at room temperature for 72 h by stirring daily. After filtration, the resulting extract was evaporated (under temperature below 40 °C and reduced pressure) to remove the solvent, and it was then lyophilized and stored at −20 °C until the beginning of the experiments.

### 2.4. Formulation of Topical Preparation

The cream base that consists of the oil phase and the aqueous phase was prepared according to the following procedure. First, the oil phase components containing 15 g of spermaceti and 60 g of liquid paraffin were weighed, and were then placed in a container and heated to 75 °C in a *bain*-*marie* to thoroughly melt them, and a smooth solution was achieved. The aqueous phase contained 25 g of deionized distilled water at 75 °C in a *bain*-*marie*, to which 0.15 g of methylparaben was added as a preservative, and after dissolution, 1 g of borax was added as an emulsifier and then mixed. After both the two phases were cooled, the aqueous phase was added to the oil phase and mixed slowly. The stirring was continued until the cream base was obtained at room temperature. After neutralization, the cream base of NaOH (pH 6.2) was formed, and the lyophilized extract of the CC was dissolved in a small amount of ethanol and added to the cream base to obtain ratios (*w*/*w*) of 2 and 4% and 8%.

### 2.5. Inflammation and Nociception Assessment

#### 2.5.1. Animal

Seventy-two male Sprague-Dawley rats (130–170 g) were obtained from the animal house and research center of AJUMS, Ahvaz, Iran. They were fed with rodent laboratory chow *ad libitum* and had free access to water. The experimental design was approved by the Institutional Animal Ethical Committee of Ahvaz Jundishapur University of Medical Science (IR.AJUMS.REC.1395.649) and conducted according to the NIH Guide for Care and Use of Laboratory Animals.

#### 2.5.2. Carrageenan-Induced Acute Inflammatory Model

The carrageenan-induced edema in a rat hind paw was carried out to evaluate the topical anti-inflammatory effect of the preparations [24]. The solution of carrageenan 1% in normal saline was prepared freshly, immediately before experiments. Edema was induced by an intraplantar injection of 100 μL carrageenan solution into the right-hind paws of each animal of all the groups, except for group I. Hydrocortisone ointment 1% was used as an anti-inflammatory reference drug. The rats were randomly assigned to the following groups: I (saline + cream base) as the saline group, II (carrageenan + cream base) as the negative control, III (carrageenan + CC 2%), IV (carrageenan + CC 4%), V (carrageenan + CC 8%), and VI (carrageenan + Hydrocortisone ointment 1%) as the positive control. All preparations in an amount of 0.3 g were used half an hour before the injection of carrageenan (1%, 100 µL, intraplantar injection) in the plantar surface of the right hind paw and it was gently rubbed 50 times with the index finger. Hydrocortisone ointment 1% and the cream base were applied in a similar way. The edema value was measured by a plethysmometer (Ugo Basile, Milan, Italy) prior to the intraplantar injection of carrageenan and at 1, 2, 3, and 4 h post-carrageenan injection. At the end of the experiment, the rats were euthanized and subplantar tissue of the carrageenan-injected paw was dissected. All tissue samples were weighed and homogenized with radioimmune precipitation assay (RIPA) buffer, and after that, the homogenates were centrifuged at 3000 rpm for 10 min at 4 °C and the supernatants were maintained at −80 °C for cytokines assessment.

#### 2.5.3. Formalin Test

To evaluate the antinociceptive effect of the cream, the formalin test was used according to the method described by Dubuisson and Dennis (1977) [25]. On the plantar surface of the right hind paw, 0.3 g of cream containing 2–8% of CC was applied and gently rubbed 50 times with the index finger. Cream base and methyl salicylate 30% were used as the control and reference drug, respectively. Thirty minutes later, 50 µL of 2.5% formalin was administrated by an intraplantar injection into the dorsal right hind paw of rats. The number of flinching events of the paw was monitored between 0–5 min (first phase) and 15–60 min (second phase) after the injection of formalin. To evaluate the local effect of cream, it was administrated on the right hind paw and 30 min later, formalin was injected into the left hind paw then, and the flinching number was recorded.

#### 2.5.4. Involvement of Opioid Receptors in the Peripheral Antinociceptive Effect of CC Cream

To determine the role of opioid receptors in the local antinociceptive effect of the CC cream, naloxone, a non-selective opioid antagonist, was used. Naloxone (20 μg/paw, i.pl.) was injected 30 min before the formalin injection [26].

### 2.6. Cytokine Assay

The tissue levels of IL-6 and TNF-α were estimated by using the commercial ELISA kit (Diaclone Research, Besancon, France) according to the manufacturer’s recommendations. The absorbance of all samples was read at 450 nm. The levels of tissue cytokines were expressed as pg/g paw tissue.

### 2.7. Skin Irritation Test

Twelve rats were divided into two groups (six rats in each group), including control and test groups. The hair was shaved behind the animal and the rats were kept in separate cages. For seven days and once a day, 50 mg of cream was placed on a 4 cm^2^ area. The area was covered with a cotton bandage, and any sensitivities were assessed and graded (A. No edema, B. Slight erythema, C. up to severe edema, D. Moderate erythema, E. Severe erythema) [27].

### 2.8. Statistical Analysis

Data were expressed as mean ± SD. Comparisons between means of different groups were carried out using one-way analysis of variance (ANOVA) followed by Tukey’s post hoc test and repeated measures ANOVA followed by Dunnett’s test. The level of significance was taken as *p* < 0.05. GraphPad Prism v. 5 (GraphPad software Inc., La Jolla, CA, USA) was used to carry out all statistical tests.

## 3. Results

### 3.1. Effect of Topical CC Cream on Carrageenan-Induced Inflammation

**Figure 1:**
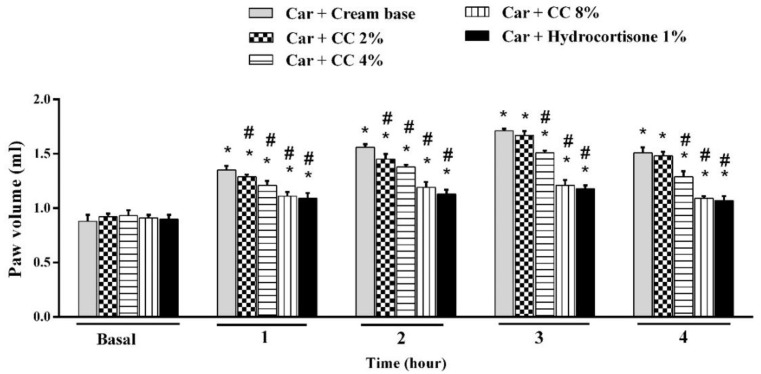
Change in paw edema at t = 1, 2, 3, and 4 h after carrageenan administration (0.1 mL of 1%, intraplantar injection). Data are expressed as mean ± SD. Car: carrageenan 1%, CC: *C. colocynthis* cream. (n = 6). * *p* < 0.05 vs. basal volume before carrageenan injection and # *p* < 0.05 vs. carrageenan group. Repeated measures ANOVA followed by Dunnett’s test.

Repeated measures ANOVA analysis of paw volume indicated that the carrageenan injection significantly increased the paw volume compared with the basal volume before the carrageenan injection (*p* < 0.001). The topical preparation of CC 2–8% significantly decreased the paw volume compared with the carrageenan group during the experiment, in a dose-dependent manner (effect of group *p* < 0.0001, of time *p* = 0.0001 and interaction effect *p* < 0.0001) (Figure 1).

### 3.2. Effect of Topical CC Cream on Cytokines Level

**Figure 2:**
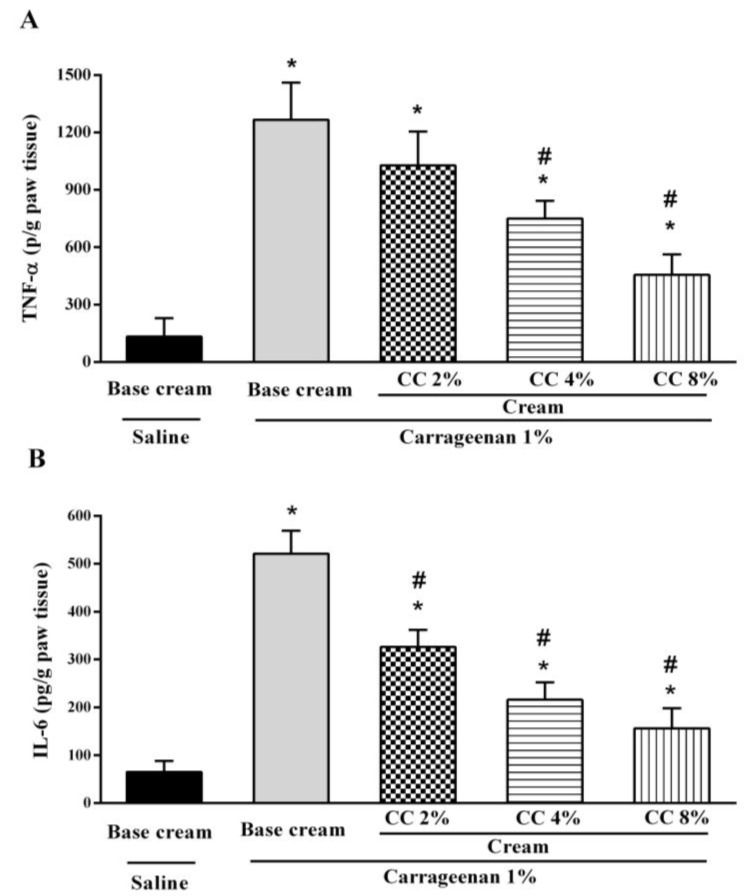
Effect of topical administration of *C. colocynthis* (CC) cream (2–8%) on the levels of TNF-α (**A**) and IL-6 (**B**) in rats 4 h after carrageenan-induced hind paw edema (0.1 mL, 1%, intraplantar injection). Data are expressed as mean ± SD (n = 6). * *p* < 0.05 vs. saline group and # *p* < 0.05 vs. carrageenan group. One-way ANOVA followed by Tukey’s test.

As shown in Figure 2A,B, carrageenan significantly increased the TNF-α and IL-6 concentration in the hind paw 4 h post-carrageenan injection compared with the saline group. The topical administration of CC cream (2–8%) significantly reversed the changes in the level of TNF-α and IL-6 due to carrageenan-induced edema, in a dose-dependent manner (*p* < 0.01).

### 3.3. Effect of Topical CC Cream on Formalin-Induced Nociception

**Figure 3:**
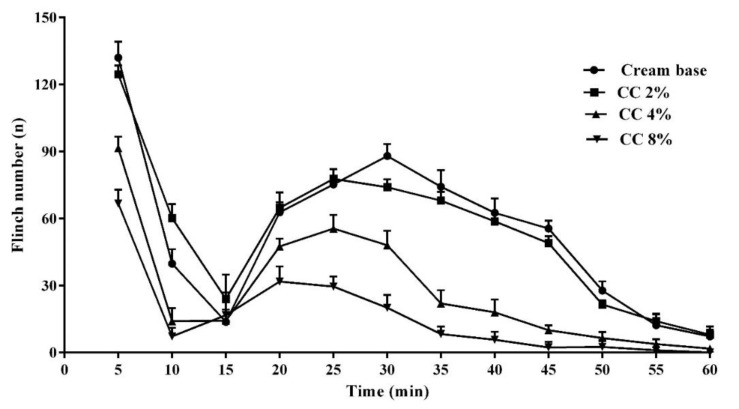
Time course of flinching induced by formalin (2.5%, 50 µL) in rats treated topically with *C. colocynthis* (CC) cream 2–8%. Values represent the mean ± SD.

**Figure 4:**
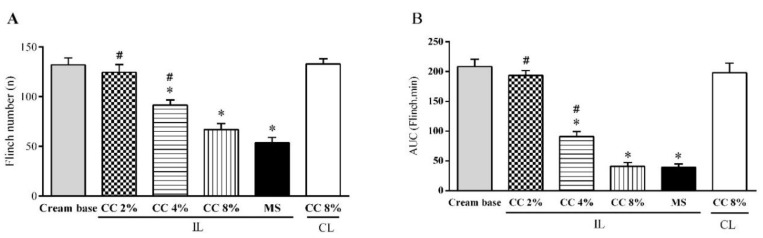
Antinociceptive effect of topical administration of *C. colocynthis* (CC) cream on the first phase (**A**) and the second phase (**B**) of the formalin test in the rat. Values represent the mean ± SD (n = 6). * *p* < 0.05 vs. cream base and # *p* < 0.05 vs. methylsalicylate (MS). One-way ANOVA followed by Tukey’s test.

**Figure 5:**
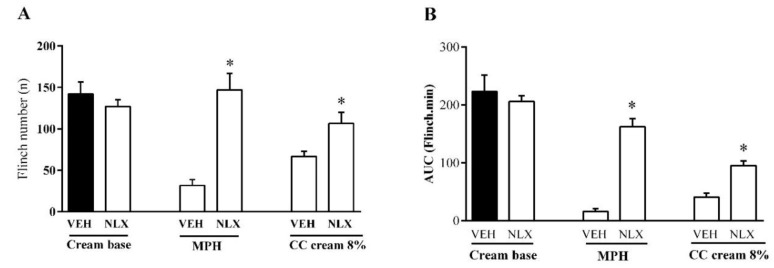
Effect of the intraplantar injection of naloxone on the antinociceptive activity of *C. colocynthis* (CC) cream in the formalin test. Naloxone (NLX, 20 μg/paw, i.pl.) was injected and then the antinociceptive effect of morphine (MPH, 25 μg/paw, i.pl.) and CC cream (8%) was evaluated in the first phase (**A**) and the second phase (**B**) of the formalin test. Values represent the mean ± S.D (n = 6). * *p* < 0.05 vs. respective treatment. One-way ANOVA followed by Tukey’s test.

The injection of formalin into the hind paw produced a biphasic nociceptive response (Figure 3). The administration of 2–8% CC cream 30 min before the formalin injection dose-dependently inhibited both the first and the second phase of the formalin test (*p* < 0.05). In both phases of the formalin test, methyl salicylate significantly inhibited pain responses and there was no significant difference between the antinociceptive effect of methyl salicylate 30% and CC cream 8% (Figure 4A,B, *p* < 0.05). The administration of naloxone (20 μg/paw, i.pl.) reversed the local antinociceptive effects of CC cream (8%) and morphine (25 μg/paw, i.pl.) on both phases of the formalin test (Figure 5A,B, *p* < 0.05).

### 3.4. Skin Irritation Test

The result showed that the topical administration of CC cream (2–8%) did not induce any allergic symptoms such as inflammation and irritation in rats up to seven days after application.

## 4. Discussion

The finding of this study revealed that the topical application of CC cream markedly suppressed the hind paw swelling induced by carrageenan and inhibited the formalin-induced nociception, which confirmed the anti-inflammatory and analgesic activity of this formulation. In addition, the evaluation of the possible mechanisms suggests that the suppression of pro-inflammatory mediators and activation of opioid receptors may be involved in these effects.

Inflammation, as the most important response of a damaged tissue which is observed in many inflammatory disorders, is characterized by redness, edema, heat, and pain at the site of injury [28]. During the inflammatory process, the sensitivity of nociceptors is heightened and the threshold of pain is decreased [29]. The tissue inflammation is mediated by the activation and infiltration of leukocytes into the damaged region and the release of biochemical and pro-inflammatory mediators, including bradykinin, prostaglandin E2 (PGE2), tumor necrosis factor-α (TNF-α), interleukin (IL-1β), and interleukin 6 (IL-6) (6). Bradykinin, PGE2, TNF-α, and IL-1β are able to induce hypernociceptive effects which lead to lowering the threshold for pain and are involved in inflammatory hyperalgesia [30,31].

Carrageenan-induced paw edema is a reproducible model that can be extensively utilized for evaluating the anti-inflammatory activity of novel compounds. A carrageenan injection into the hind paw induces a biphasic inflammation pattern, which in the first phase mediators such as histamine, leukotrienes, serotonin, cyclooxygenases, and kinins, is increased. Meanwhile, in the delayed phase, the elevation of prostaglandins, arachidonic acid metabolites, and the neutrophil influx have been observed [24]. The effect of anti-inflammatory agents is evaluated by measuring changes in paw swelling due to the injection of carrageenan into the hind paw.

The present study is supported by many experiments showing that an intraplantar injection of carrageenan into the hind paw of rats induces a severe inflammatory response and results in paw swelling [24]. However, the topical application of CC cream clearly ameliorated paw swelling induced by carrageenan at each time point (1, 2, 3, and 4-h post-carrageenan injection), and the anti-inflammatory effect of CC 8% was comparable to that of hydrocortisone ointment 1%. These findings are in agreement with previous studies. Marzouk et al. (2010) showed that immature fruit of CC aqueous extract (1 and 4 mg/kg, intraperitoneally) suppressed both phases of inflammation induced by a carrageenan rat model of paw edema and the maximum anti-edematous effect was at 6 h after the carrageenan injection [10]. Also, in another study, they reported that all fractions of CC fruit (orally), including chloroform, petroleum, ether, methanol, acetone, and ethyl acetate extract, displayed a marked anti-inflammatory effect in an experimental model [2]. Overall, our result confirmed that the topical administration of CC exhibited anti-edematous activity similar to systemic administration in experimental animal models of inflammation.

Several studies have shown that an intraplantar injection of carrageenan leads to an increase in the expression and release of various cytokines, for instance, TNF-α and IL-1β, which in turn cause the release of further pro-inflammatory mediators, including IL-6, kinins, leukotrienes, arachidonic acid metabolites, and reactive oxygen species [32,33]. These cytokines are the key components of the innate immune system which activate leukocytes and provide host defenses against tissue injuries [19,34]. The present investigation showed that paw edema induced by carrageenan was synchronized by early elevation in TNF-α and IL-6 in hind paw tissue. Our finding indicated that pretreatment of carrageenan-received rats either with topical cream of CC or with hydrocortisone 1% considerably reduced the TNF-α and IL-6 levels in hind paw tissue compared to the rats treated with a cream base. Thus, the CC cream reduced the susceptibility of rats’ paw tissue to pro-inflammatory mediators. These findings confirm recent reports where CC downregulated these pro-inflammatory mediators in obesity induced by a high-fat diet (HFD) [16], diabetes mellitus [5], osteoarthritis [17], and inflammation induced by carrageenan in an animal model [2]. Akhzari et al. (2015) showed that the expression of pro-inflammatory mediators such as TNF-α, COX-2, and INOS in chondrocyte cells and monocytes/macrophages were reduced by treatment with an ethanol extract of CC [17]. Since the anti-inflammatory activity of the topical administration was equal to hydrocortisone 1%, the CC cream can be suggested as an effective agent for inflammatory disease.

In this study, the analgesic effect of the CC cream was evaluated using the formalin test in the rat. The formalin test is a highly reproducible and common in vivo model for the evaluation of the analgesic potential of drugs and possible mechanisms. In the rat, an intraplantar injection of formalin produces a biphasic pain response. The first phase (0–5 min), which is called the neurogenic phase, is related to the activation of C fibers, and the second phase (15–40 min) is due to inflammatory mechanisms and the release of local mediators and sensitization of peripheral and spinal cord neurons [35]. Analgesics have different effects on the formalin test due to their mechanism of action. Opioids and most NSAIDs can inhibit both phases of the formalin test, but acetaminophen only inhibits the second phase [36]. In this study, the ipsilateral plantar application of CC cream inhibited both phases of the formalin test in a dose-dependent manner. Morphine and methyl salicylate as reference drugs inhibited both phases of the formalin test. The antinociceptive effect of the CC cream (8%) was comparable to that of methyl salicylate cream 30%. However, the application of CC cream 8% (high dose) on the contralateral paw did not inhibit the formalin-induced nociception, indicating that the CC acted peripherally at the site of application. Our data are supported by a previous study, which showed that the oral administration CC extract reduced the number of writhing events in acetic acid-induced writhing in mice [2,10].

Opioid receptors in the peripheral nervous system and immune cells are involved in modulating pain and inflammation. Various behavioral studies using inflammatory models have shown that exogenous opioids exert an antinociceptive effect by activating peripheral opioid receptors. These receptors are expressed on small-, medium-, and large-diameter sensory neurons [37]. The activation of peripheral opioid receptors coupled to inhibitory G-proteins reduces cyclic AMP, increases K efflux, and decreases Ca^2+^ entry in the sensory nerve terminals and ultimately reduces action potential propagation and the release of excitatory neuropeptides [38]. Drugs that selectively activate peripheral opioid receptors have therapeutic potential for pain control. Therefore, in this study, the role of opioid receptors in the peripheral antinociceptive effect of CC was investigated. In this study, pre-treatment with naloxone reversed the antinociceptive effect of CC in both the first and second phases of the formalin test. It indicated that peripheral opioid receptors are involved in the antinociceptive effect of CC and at least some of the antinociceptive effects of the CC are associated with the activation of opioid receptors or increased release of endogenous opioid. Furthermore, since in the second phase of the formalin test, the analgesic effect of the extract completely was not reversed by the naloxone, other mechanisms such as the anti-inflammatory effect are probably involved in the peripheral antinociceptive effects of CC cream. The anti-inflammatory activity of CC has been revealed in various animal models [5,10]. In this study, we showed that the topical application of CC cream decreased inflammatory mediators (TNF-α and IL-6) in the carrageenan-induced paw edema model.

Since the strong analgesic and anti-inflammatory potential of flavonoids and alkaloids is well documented [39], it seems that the antinociceptive and anti-inflammatory activity of CC cream may be associated with its active ingredients, including flavonoids and alkaloids.

## 5. Conclusions

For the first time, our investigation showed that the topical of CC fruit exhibits marked anti-inflammatory and antinociceptive activities. The anti-inflammatory and analgesic effects of CC are significant and are comparable to steroidal anti-inflammatory agent hydrocortisone and NSAIDs, respectively. These therapeutic benefits of CC could probably be related to the opioid receptors and its ability to suppress the pro-inflammatory mediators. Thus, topical CC preparations are promising as a topical anti-inflammatory and analgesic agent and have the potential application as a complementary therapy for the treatment of inflammatory disorders. However, further pharmacological studies are needed for their clinical application.
